# Annual Report of the State Veterinarian of Pennsylvania

**Published:** 1899-05

**Authors:** 


### NEW PUBLICATIONS.

### ANNUAL REPORT OF THE STATE VETERINARIAN OF PENNSYLVANIA.

THIS work, just issued, is a brief but concise report of the veterinary sanitary work done in the Keystone State during 1898. The subjects of rabies, anthrax, cornstalk disease, hog-cholera, tuberculosis, glanders, black quarter, abortion, milk-fever, Texas fever, lung-worm disease, dysentery in calves, are all reported upon according to their importance in the State and the methods of control, etc. The expense entailed and the results obtained in defensive measures, educational quarantine, remedial and protective, are fully dwelt upon, and we trust that every veterinarian interested in veterinary sanitary work will be privileged to review what can be accomplished by the expenditure of a moderate amount of money when directed along conservative, intelligent, and practical lines. It is a strong commendation of the valued services rendered the Commonwealth by an efficient officer.

